# Recent advances in managing benign prostatic hyperplasia: The Rezūm System

**DOI:** 10.12688/f1000research.15851.1

**Published:** 2018-12-10

**Authors:** Dominique Guelce, Dominique Thomas, Dean Elterman, Bilal Chughtai

**Affiliations:** 1 Urology, Weill Cornell Medical Center, New York, NY, 10065, USA; 2 Urology, University of Toronto, Toronto, Ontario, M5T2S8, Canada

**Keywords:** Rezum; Benign Prostatic Hyperplasia, Lower Urinary Tract Symptoms

## Abstract

Benign Prostatic Hyperplasia is a common condition that affects 50% of men in their 50 ^th^ decade. There have been many advances in the treatment of this condition, which aim to improve the patient’s quality of life. A new treatment that shows promising results is the Rezūm System, a water vapor therapy for BPH. We present the most current literature on this therapy.

## Introduction

The Rezūm System uses thermal energy through the convection of radiofrequency water vapor into the prostate. The system is composed of a radiofrequency generator and transurethral probe, which delivers the water vapor at a constant dose of 208 calories (vapor temperature of 103°C). Saline flush irrigation can also be delivered through the probe to enhance visualization and cool the urethral surface. At the end of the probe, there is a small lens for cystoscopic visualization and an 18-gauge polyetheretherketone needle with 12 holes, each 30° apart, allowing circumferential distribution of the water vapor ^1^. Water vapor is injected into the targeted lobe, which heats to around 70°C, and disrupts the cell membranes, leading to cell death. Multiple injections, each lasting 9 seconds, may be applied to each lobe of the prostate ^2^. The injections begin 1 cm from the bladder neck and progress 1 cm each with a distal endpoint of the verumontanum. Because convective energy is being used rather than a conductive one, as in thermo-ablation, a temperature gradient is not established. The moving vapor remains in the targeted area passing between cells, unable to pass between zones because of the vapor’s low density ^3^. This decreases the risk of complications to neighboring structures and theoretically confines the steam to the obstructing transition zone.

A literature search was conducted by searching PubMed for publications about the Rezūm System. A total of 9 articles were included in the review for their content, and 4 articles were removed because they were reviews or commentaries.

## Commentary

The first pilot study ^4^ assessed the ability of the Rezūm System to treat lower urinary tract symptoms (LUTSs) secondary to benign prostatic hyperplasia (BPH). A total of 65 men underwent the Rezūm System procedure. The inclusion criteria required that the men be over 45 and have an International Prostate System Score (IPSS) over 15, maximum flow rate (Qmax) under 15 mL/second, post-void residual volumes under 300 mL, and prostatic volumes between 20 and 120 mL. The effectiveness of the procedure was assessed quantitatively by measuring these values at 1 week and 1, 3, 6, and 12 months. By the 3-month time point, there was a decrease in the average IPSS of 13.4 points, a 60% improvement from baseline. The average Qmax had increased by 4.7 mL/second at 3 months, an increase by 85%. Furthermore, at 12 months after the procedure, IPSS improved by 56% ( *p* <0.001) while Qmax had an 87% improvement ( *p* <0.001). Post-void residual volume was reduced by 20% at the 3-month time point and by 12 months had remained reduced by 12%. Prostate volume showed a 28.9% reduction at the 6-month time point. Furthermore, men had an overall 61% improvement in quality of life at 12 months. Sexual function was preserved across all time points. Maximal improvement was noted in nearly all endpoints by the 3-month time point and was maintained to the 12-month time point, remaining durable even 2 years post-operatively.

A study by Mynderse *et al*. evaluated the physical effects and severity of the lesions to the prostatic tissue ^5^. This was observed under serial gadolinium-enhanced magnetic resonance imaging 6 months after the Rezūm System procedure ^5^. The lesions were characterized by size and by “time course of the ablative lesion resolution”. Change in prostate size was also monitored. Time points of 1 week and 1, 3, and 6 months were used. One week after treatment, the lesions were noted to be an average size of 8.2 cm ^3^. By month 1, the lesions had resolved by 58.5%, and by months 3 and 6, the lesions were resolved by 91.5% and 95.1%, respectively. At the 6-month time point, there had been an average decrease in prostate tissue volume of 28.9%.

This was followed by a study by McVary and Roehrborn, who conducted a 2-year multi-center double-blind randomized controlled trial (RCT) comparing the Rezūm System with a sham procedure ^6^. The sham procedure involved a cystoscopy and simulated treatment sounds. A total of 197 men participated in the study. The inclusion criteria included age over 50, IPSS of at least 13, Qmax between 5 and 15 mL/second, and prostate size between 30 and 80 mL. A total of 136 men were randomly assigned to the Rezūm group and 61 were assigned to the sham group. The groups were blindly compared after 3 months. The Rezūm System decreased IPSS by 11.2 points compared with a 4.3 drop in the sham procedure group. The peak flow rate for the treatment group increased by 6.2 mL/second by the 3-month time point as well. There was no noticeable difference in treatment outcomes for those with or without a median lobe in the treatment group. The treatment arm was followed for 12 months after initial treatment and these results were sustained. When compared with sham, patients treated with Rezūm improved whether or not the median lobe was treated. However, in a subsequent subanalysis of this data set, patients with treated median lobes had a greater flow and lower IPSS at 12 months compared with those who had a median lobe that was identified and not treated (change of +6.5 mL/second versus +2.8 mL/second and symptom score decrease of −12.4 versus −9.9). There was no change in ejaculatory function whether or not the median lobe was treated. Treating the median lobe, when present, is better than not treating it.

The Rezūm System has shown not only that it is effective in resolving LUTSs but also that it is safe. All 129 patients seen by a single surgeon in the study by Mollengarden *et al*. ^7^ successfully completed the procedure, and the most common adverse events were urinary tract infections (17%) and transient urinary retention (14%). However, because the maximal effect of the Rezūm System is experienced after 3 months, it is difficult to determine whether these urinary tract infections were due to symptomatic infection or post-operative LUTSs with bacteriuria ^7^. On top of significant improvements in quality of life and post-void residual volume, patients from the study by Darson *et al*. experienced no *de novo* erectile or ejaculatory dysfunction ^1^. However, typical surgical procedures for BPH may have risks for erectile dysfunction. In fact, ejaculatory bother scores improved over baseline from 12 months to 36 months and 32% of patients noted an improvement in erectile function in the study by McVary and Roehrborn. The Rezūm System has shown its potential as a minimally invasive alternative to medication or other transurethral procedures. Since the early studies assessing safety and efficacy, studies comparing Rezūm with other treatments have been examined.

A direct comparison of the Rezūm System with a medication-based approach has never been conducted. However, McVary *et al*. carried out an indirect comparison of the two by comparing the results of their own Rezūm System RCT ^6^ and those of the National Institute of Diabetes and Digestive Kidney Diseases (NIDDK) 1995 Medical Therapy of Prostatic Symptoms (MTOPS) trial ^8, 9^. Three-year outcomes for both medical treatment and the Rezūm System were compared with each other, albeit through two separate studies conducted over a decade apart. In the MTOPS trial, 3,047 men participated. In a 1:1:1:1 ratio, they were assigned to placebo, an alpha blocker (doxazosin), a 5-alpha reductase inhibitor (5-ARI) (finasteride 5 mg), or a combination therapy of the latter two. The protocol followed the patients annually for 5 years. Patients from the MTOPS trial who met the inclusion criteria for the RCT by McVary and Roehrborn were used in this study. This allowed the use of 1,140 subjects: 367 were from the doxazosin treatment, 390 from 5-ARI, and 383 from the combination therapy. In total, 7 patients from the Rezūm procedure were removed because they had an IPSS that exceeded the maximum for the MTOPS trial. In terms of subjective patient data, the Rezūm System outperformed either medical treatment in IPSS reduction (2.6 points better than doxazosin and 5.2 points better than finasteride) and combination therapy had similar improvements in IPSS to those seen with Rezūm. More objectively, the Rezūm System resulted in greater increases in Qmax ( *p* <0.001) than all three medical treatments up to the 3-year time point. However, the Rezūm System was outperformed by combination therapy in terms of decreasing and sustaining a lowered post-void residual volume for up to 3 years ( *p* <0.001). Table 1 gives a summary of all the studies described in this review.

**Table 1.** List of articles summarized.

Source	Topic
Dixon *et al*. ^4^ (2015)	Pilot data for the Rezūm System and how the system works
Mynderse *et al*. ^5^ (2015)	Determine the physical effects of water vapor convection on tissue and evaluate how well the lesions resolve
Mollengarden *et al*. ^7^ (2017)	A retrospective look at a single surgeon’s results with the Rezūm System after performing the procedure on 129 patients
Darson *et al*. ^1^ (2017)	Data accrued from seven urologists using the Rezūm System for the treatment of benign prostatic hyperplasia (BPH)- associated lower urinary tract symptoms (LUTSs)
Chung and Woo ^2^ (2018)	A look at the up-and-coming minimally invasive treatments for BPH
McVary and Roehrborn ^6^ (2018)	Three-year outcomes for the treatment of patients with BPH-associated LUTSs compared with those undergoing a sham procedure
Gupta *et al*. ^8^ (2018)	Three-year outcomes for the treatment of patients with BPH-associated LUTSs compared with those undergoing medication-based treatment in the Medical Therapy of Prostatic Symptoms (MTOPS) trial

## Conclusions

These initial data for the Rezūm System seem very promising. Rezūm has demonstrated significant improvements in American Urological Association symptom score, flow rate, and prostate volume reduction. When compared indirectly with medical therapy, Rezūm was notably superior to monotherapy. The Rezūm System has the advantage of being performed in an office setting under local anesthesia in prostates of various shapes and sizes. The Rezūm System also allows the treatment of median lobes while preserving erectile and ejaculatory function. Looking ahead, we are awaiting the results of real-world data in comparison with other minimally invasive office-based therapies. Rezūm has the potential to be a useful tool in the armamentarium of urologists treating BPH.

## Abbreviations

5-ARI, 5-alpha reductase inhibitor; BPH, benign prostatic hyperplasia; IPSS, International Prostate System Score; LUTS, lower urinary tract symptom; MTOPS, Medical Therapy of Prostatic Symptoms; Qmax, maximum flow rate; RCT, randomized controlled trial
